# Acute Neuropathic Orchalgia and Scrotalgia After Percutaneous Spinal Cord Stimulator Lead Placement: Two Cases with an Unusual Complication

**DOI:** 10.7759/cureus.1003

**Published:** 2017-01-30

**Authors:** Meng Huang, Virendra R Desai, David Ho, Richard K Simpson

**Affiliations:** 1 Department of Neurosurgery, Houston Methodist Neurological Institute; 2 Urology, Houston Methodist Hospital

**Keywords:** spinal cord stimulation, orchalgia, scrotalgia, neuropathic

## Abstract

Spinal cord stimulation is an effective adjunct to the treatment of a variety of chronic pain syndromes. Complications are relatively low in morbidity and are most often secondary to hardware malfunction/malposition. Infection and undesired dysesthesias represent only a minority of complications. Neuropathic orchalgia and scrotalgia after placement of epidural spinal cord stimulator is a previously unreported morbidity. While alarming, this condition is physiologically benign, causing no neurological or urological dysfunction. The two cases we encountered both occurred during uncomplicated percutaneous trial stimulator placement. Corticosteroid treatment and stimulator activation facilitated resolution of the dysesthesia and allowed completion of the trial in one case, while the other case was refractory and resulted in termination of the trial.

## Introduction

Dorsal column stimulation is a valuable adjunct in the treatment of many types of chronic pain syndromes including failed back surgery syndrome/post laminectomy syndrome, complex regional pain syndrome, small-fiber peripheral neuropathy, vascular claudication, and visceral pain [1-6]. The majority of complications are hardware related, and undesirable sensory dysesthesias account for only a minority of revisions and explantation procedures [4, 7-9]. To the best of our knowledge, acute referred orchalgia and scrotalgia related to stimulator placement have never been reported in literature. Here we report two cases of routine uncomplicated thoracic percutaneous spinal cord stimulator leads under conscious sedation with this unusual complication. Informed consent was obtained from the patients for this study.

## Case presentation

### Case 1

**Figure 1:**
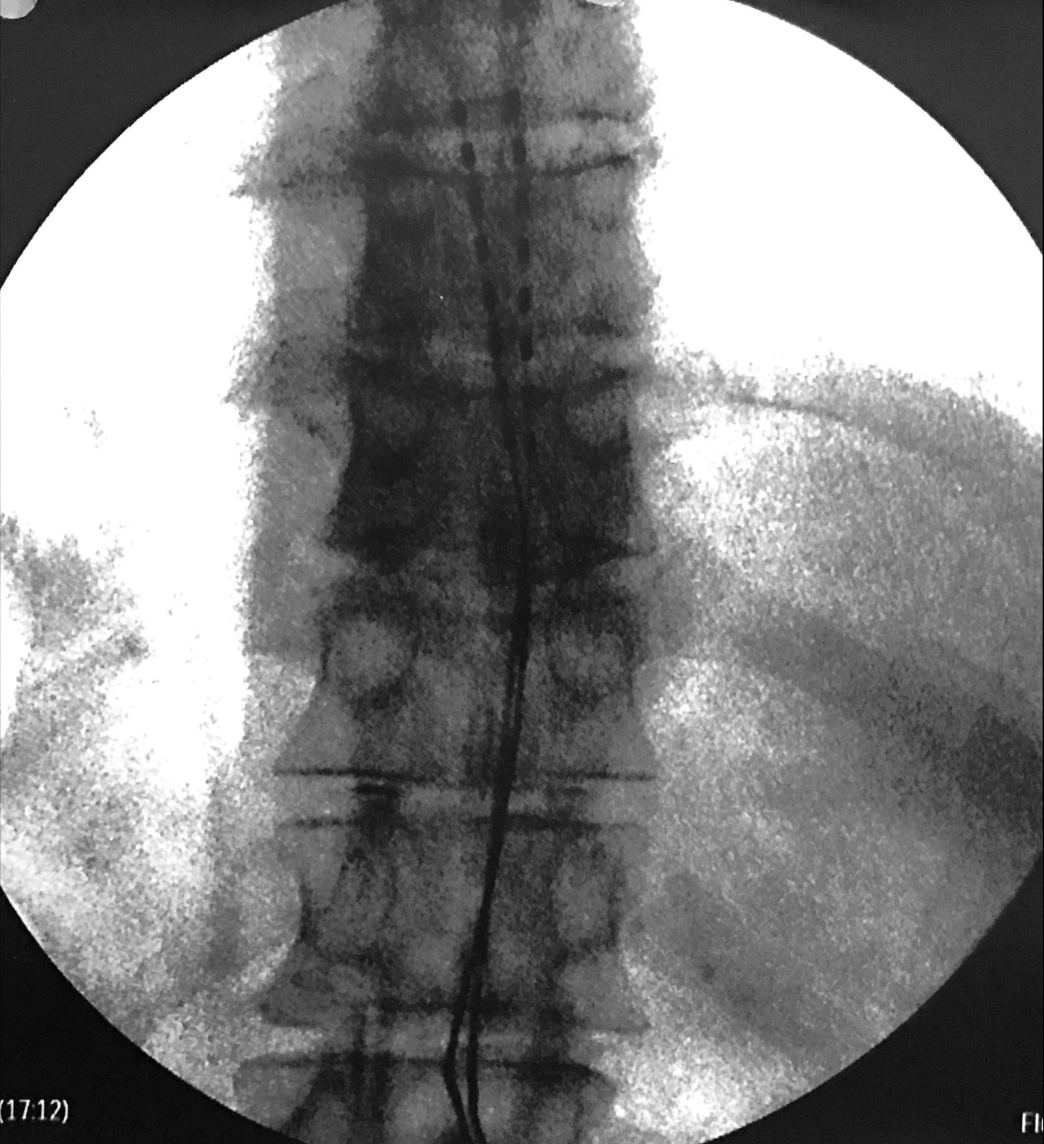
Case 1: Intraoperative final anteroposterior (AP) fluoroscopic image. The twelfth rib is the inferior-most rib.

A 59-year-old male with a history of multilevel lumbar laminectomy was referred to us for trial and permanent placement of epidural spinal cord stimulator for failed back surgery syndrome with persistent low back pain and lumbar radiculopathy. He was cleared by formal neuropsychiatric testing and subsequently underwent routine trial with two Vectris Surescan (Medtronic Sofamor Danek USA, Inc. TN, USA) percutaneous eight contact epidural electrodes under conscious sedation. Saline push technique with Omnipaque epidurogram (GE Healthcare, CT, USA) confirmed epidural placement. Intraoperative fluoroscopy showed both electrodes at the desired levels rostrally at the bottom of the vertebral body of T8, spanning the entirety of T9 and caudally ending at the top of T10. One electrode was slightly off to the right of midline. Sedation was lightened and the system was activated for intraoperative testing. The patient did not complain of any dysesthesias or undesired stimulation and confirmed excellent coverage of his pain. No abnormal parameters were noted and all contacts were working normally during intraoperative interrogation. Final anteroposterior (AP) X-ray at the end of the procedure confirmed proper placement, and the electrodes were secured to the skin in the usual fashion with silastic anchors and silk sutures (Figure 1).

Immediately postoperatively in the recovery unit, he complained of severe and distressing sharp testicular and scrotal pain in the abscence of neurological deficits. He was placed on high dose corticosteroids, which improved his pain. He was evaluated by urology service and was found to have no evidence of intrinsic testicular or scrotal pathology. His bladder function remained normal without urinary retention. His stimulator was turned on the next day, and his overall pain and discomfort markedly improved, and therefore, no further workup or imaging was obtained. He was discharged home for the completion of his trial and eventually returned for permanent placement with a laminectomy style paddle electrode with an open mini-laminectomy approach. His permanent placement postoperative course was unremarkable and he ultimately reported therapeutic stimulation. 

### Case 2

**Figure 2:**
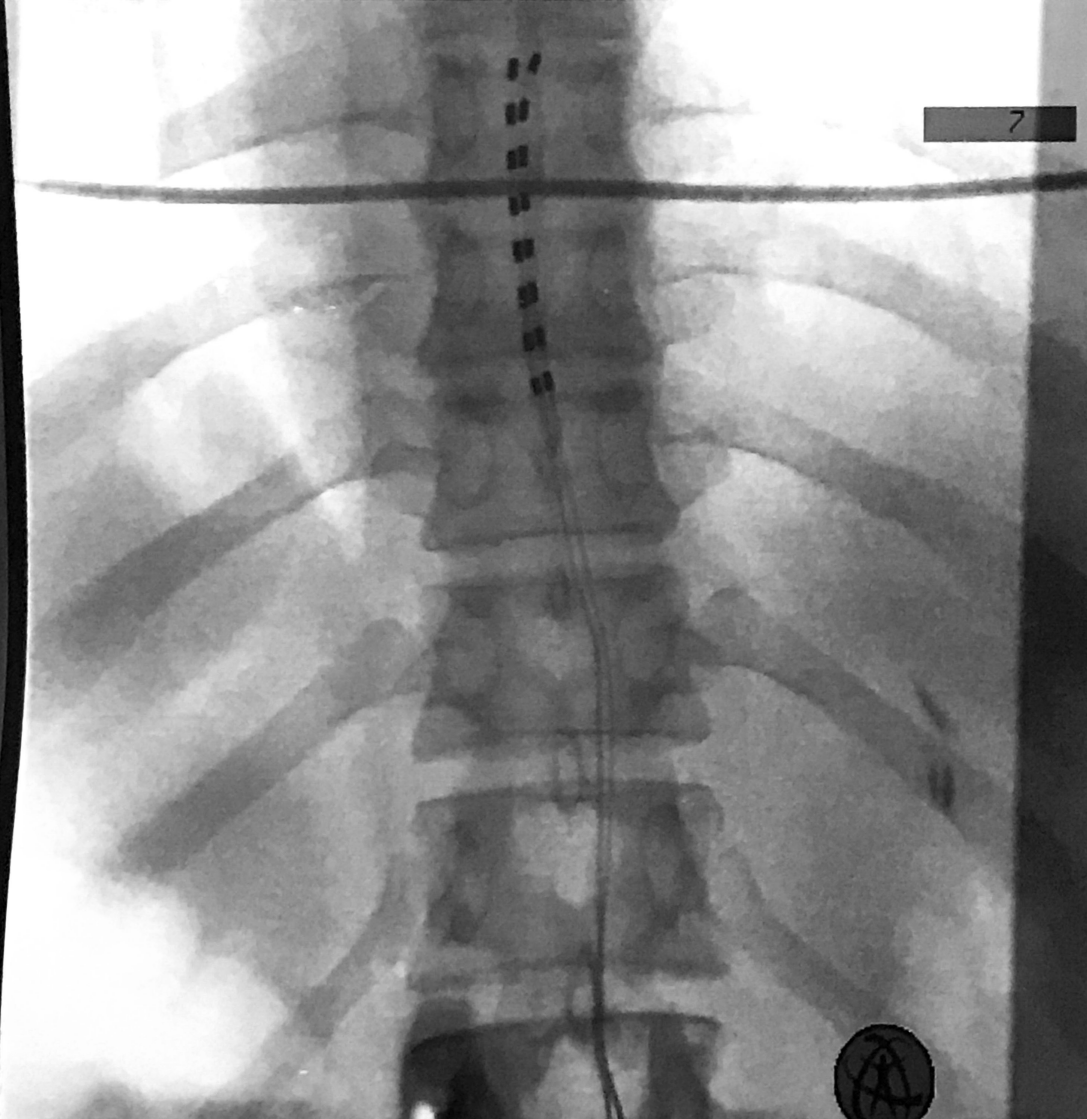
Case 2: Intraoperative final AP fluoroscopic film. The twelfth rib is the inferior-most rib.

A 34-year-old male with history of two prior lumbar spine surgeries presented to our clinic with complaints of back and left leg pain that was refractory to aggressive medical measures and failure of surgical decompression. He was deemed a good candidate for percutaneous spinal cord stimulator placement after routine preoperative neuropsychiatric testing, and thus underwent a trial placement with two 8-contact epidural electrodes (Octrode Trial, St. Jude Medical, MN, USA) using the same technique described above. Both electrodes were seated with the top portions of the electrodes placed at the T7-T8 disk space, with one electrode directly in the midline and the other just to the left of midline. During intraoperative testing, the patient confirmed paresthesias covering his areas of pain without reporting any dysesthesias. No abnormal parameters were noted and all contacts were working normally during intraoperative interrogation. These electrodes were then secured to the patient’s skin and final X-rays confirmed placement as described above (Figure 2).

**Figure 3:**
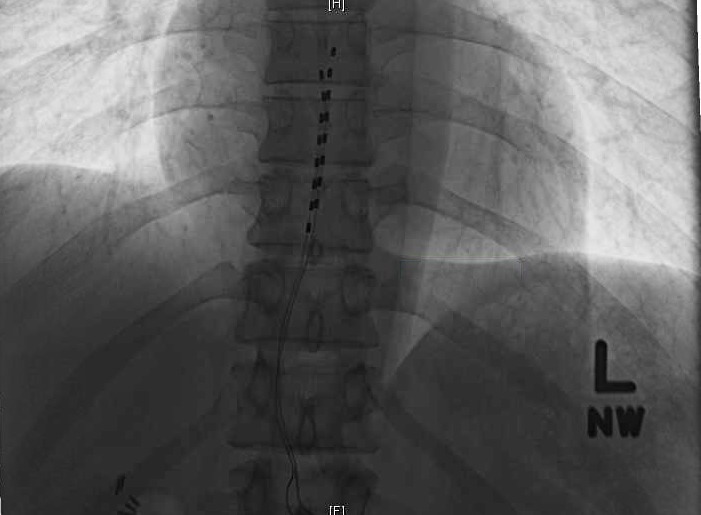
Case 2: Postoperative AP X-ray showing minor lead shift without lateral migration.

**Figure 4:**
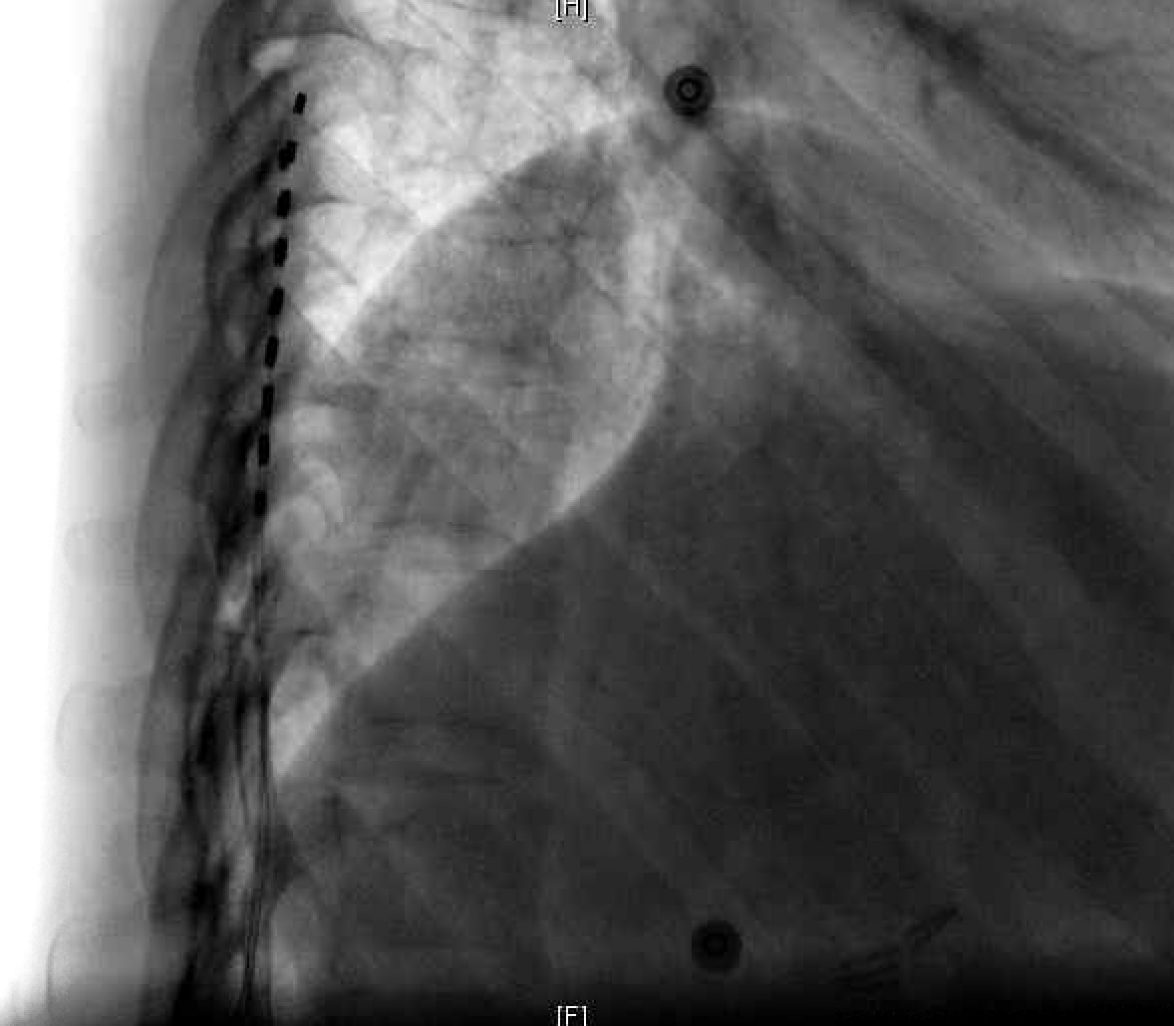
Case 2: Postoperative lateral X-ray showing no evidence of ventral migration.

The patient was transported to the recovery room where he began complaining of severe and alarming scrotal pain. He was given high dose corticosteroids, high dose patient-controlled analgesia pump, and neurontin, but he experienced no relief of his pain. His neurologic examination was otherwise normal and bladder function remained normal. He underwent a complete urologic evaluation, which ruled out primary pathology that would have been attributable to his pain. Activation of stimulation resulted in aggravation of his pain, and AP and lateral radiographs were acquired. These revealed minor inferior lead shift without lateral or ventral migration (Figures 3-4).

Due to the refractory nature of his pain, the patient elected for termination of his trial, after which the patient experienced immediate relief of his scrotal pain with no adverse neurologic sequelae.

## Discussion

Complications related to spinal cord stimulators can be separated into two categories: hardware and biological. Hardware complications are those attributable to electrode lead migration or fracture/malfunction and pulse generator migration, malfunction, or local site pain. Biologic complications encompass wound dehiscence, superficial or deep infection, and undesired paresthesias and dysesthesias. The average adverse event rate reported in the literature is 34–38%, with the majority of these attributable to hardware complications including lead migration (22.6%) and subsequent need for revision [4, 7-8]. In a recent series of 238 patients, Hayek, et al. reported a complication rate of 2.6% related to undesired dysesthesias, which in all cases forced explantation [9].

Stimulator implant related thoracic radiculopathy has been reported in the literature [10]. In our experience, undesired thoracic radiculopathy and/or referred abdominal visceral pain are not uncommonly encountered at the time of implantation and are attributable to lateral and ventral malposition of leads, respectively. This is easily detected by patient-reported discomfort during intraoperative testing and subsequently confirmed by anteroposterior and lateral radiography showing lateral and ventral malposition of leads, respectively.

Despite successful intraoperative testing and final intraoperative X-rays confirming proper electrode placement, we encountered two cases of severe neuropathic orchalgia and scrotalgia. To the best of our knowledge, this character of undesired stimulation has never before been reported in the literature, and the cause of the underlying pain remains unclear to us.

In the first case, the undesired orchalgia/scrotalgia responded to corticosteroids and stimulator activation. This argues against lateral migration-induced thoracic radiculopathy given that activation of stimulation improved symptomatology. Similarly, visceral referred pain and undesired abdominal muscle activation from ventral lower thoracic stimulation should have worsened with stimulator activation. His neurological exam was otherwise normal, so our suspicion for epidural hematoma causing significant mass effect was low. In the second case, the pain was refractory to all measures and made worse with stimulation activation, so our initial suspicion was lateral and or ventral migration of the leads; however, subsequent AP and lateral X-rays revealed only minor caudal lead shift without lateral or ventral migration. His neurologic exam was normal otherwise, and therefore our suspicion of epidural hematoma with significant mass effect was low. With removal of the leads, his pain resolved, and his neurological status remained intact.

From a product standpoint, these were FDA-approved standard and routinely used electrodes from both companies with no obvious external signs of hardware abnormality. Intraoperative interrogation was routine without abnormal electrical parameters. In hindsight, we unfortunately did not think to send the explanted items back to the company for technical evaluation.

## Conclusions

Acute referred orchalgia and scrotalgia is a previously unreported complication related to spinal cord stimulation that can be painfully incapacitating and result in trial termination. Urologic evaluation revealed no primary testicular or scrotal etiology, and bladder function and neurologic status remained unchanged from baseline. In our first case, corticosteroids and stimulator activation resulted in improvement of the adverse symptom; however, in our second case, the pain was refractory to all measures and immediately relieved after removal of leads. In both cases, thoracic radiculopathy from lateral lead migration and referred visceral pain from ventral migration and stimulation were felt to be unlikely based on clinical course in the first case and postoperative radiographic evidence in the second case. The hypothetical pathophysiologic process behind these adverse events remains uncertain. When faced with this rare but distressing clinical scenario, patient reassurance, corticosteroid administration, and stimulator activation may facilitate trial salvage.
